# Improvement of rooster semen freezability and fertility rate after sericin supplementation in freezing semen extender

**DOI:** 10.5713/ab.23.0016

**Published:** 2023-05-02

**Authors:** Ruthaiporn Ratchamak, Supakorn Authaida, Wuttigrai Boonkum, Vibuntita Chankitisakul

**Affiliations:** 1 Department of Animal Science, Faculty of Agricultural, Khon Kaen University, Khon Kaen 40002, Thailand; 2 The Research and Development Network Center of Animal Breeding and Omics, Khon Kaen University, Khon Kaen 40002, Thailand

**Keywords:** Cryopreservation, Frozen-thaw Semen, Lipid Peroxidation, Thai Native Chicken

## Abstract

**Objective:**

Semen cryopreservation result in decreased sperm parameters and fertilization ability. Sericin exhibits antioxidant activity by reducing lipid peroxidation resulting from free radicals, which can potentially improve cryopreservation outcomes. The present study aimed to examine the efficacy of various sericin concentrations supplemented with a rooster semen-freezing extender on post-thaw semen quality and fertilizing ability of sperm after cryopreservation.

**Methods:**

Semen samples were collected from 40 roosters (5 reps), then were pooled, and divided into four groups by the levels of sericin supplementation (0%, 0.25%, 0.50%, and 0.75%) in a freezing extender. Semen suspensions were loaded in medium straw (0.5 mL) and cryopreserved with the traditional liquid nitrogen vapor method. Post-thawed semen was evaluated for sperm motility, sperm viability, and lipid peroxidation. Also, the fertility test was determined.

**Results:**

The results showed that supplementation of the freezing extender with 0.50% to 0.75% sericin resulted in greater total motility and progressive motility and lower malondialdehyde levels than the other groups after cryopreservation (p<0.05). However, the viability of 0.75% decreased compared with the value of 0.50% sericin supplementation (p<0.05). Moreover, the fertility and hatchability of total eggs were significantly higher in the 0.50% sericin group than in the other groups (p<0.05).

**Conclusion:**

In conclusion, 0.50% sericin is recommended as an alternative component of the freezing extender to improve cryopreserved rooster semen.

## INTRODUCTION

Rooster sperm cryopreservation is an invasive technique for genetic preservation. However, the reports of success rates are variable. The problem is mainly due to the chicken sperm plasma membrane containing high amounts of polyunsaturated fatty acids (PUFAs), which tend to make sperm cells susceptible to harmful reactive oxygen species (ROS) [1]. In addition, their membranes having a high cholesterol to phospholipids (C/PL) ratio allows for the membrane to remain fluid during the freezing process [2]. However, this ratio in roosters is quite low (0.25 to 0.30) [3]. The characteristics of a lower C/PL ratio and higher quantities of PUFAs in the sperm plasma membrane make poultry sperm cells extremely vulnerable to lipid peroxidation due to oxidative stress during cryopreservation processing, resulting in membrane damage, decreased motility, and loss of fertilizing capacity [4]. To improve rooster cryopreserved semen quality, several antioxidant substances are necessary to supplement the freezing extender to decrease the ROS that occur during the freezing process, leading to improved sperm viability after thawing as reviewed by Partyka and Niżański [4].

Sericin is a protein that contains high levels of hydroxyl-containing amino acids (serine and threonine) derived from silkworms. It has been documented that the presence of amino acids positively affects cryo-resistance [5]. Sericin exhibites antioxidant activity by reducing the lipid peroxidation resulting from free radicals [6]. Previous reports on its antioxidant properties indicate that sericin acts as a cryoprotective agent during bovine embryo freezing [7,8]. Several studies have shown that sericin can improve frozen-thawed semen, for instance, human, rabbit, boar, and dairy bull semen, by protecting sperm from oxidative stress [9–12]. However, sericin has not been studied in poultry with different cryopreservation processing strategies from mammals [3]. Additionally, the optimal sericin dose for each species was shown to differ. For example, supplementation with 0.75% sericin was recommended for freezing extenders to improve cryopreserved boar semen [11], while supplementation with 0.25% sericin seemed to be optimal for enhancing sperm quality in dairy bull semen [12]. Therefore, the present study aimed to examine the efficacy of various concentrations of sericin supplemented with the semen-freezing extender of the rooster on post-thaw semen quality. The malondialdehyde (MDA) concentration was measured as an index of lipid peroxidation in semen samples. In addition, we examined the effect of insemination on the most effective solid-stored semen.

## MATERIALS AND METHODS

The experimental protocol was approved by the Animal Ethics Committee of Khon Kaen University (IACUC-KKU- 92/65; Reference No. 660201.2.11/644 [113]). Unless otherwise stated, all chemicals used in this study were purchased from Sigma–Aldrich Chemical Co. (St. Louis, MO, USA).

### Animals

Forty Thai native roosters (Pradu Hang Dum) that were 78 weeks of age were managed intensively in a battery cage system, with 60×45×45 cm^3^ per rooster and 16 h light/d throughout the experiment. Each rooster received approximately 130 g of commercial breeder feed for male chickens per day, and water was given ad libitum. One hundred and sixty Thai Native hens (Chee breed) that were 32 weeks of age, with egg production >80%, were used for the fertility test. The hens were housed individually, fed approximately 110 g of layer feed per day and were given water *ad libitum*.

### Rooster sperm preparation and semen dilution

Rooster semen samples were routinely collected twice a week from individual roosters in a 1.5-mL microtube using the dorso-abdominal massage method. The samples were kept at a temperature of 22°C to 25°C during transport to the laboratory for further analyses. Transport occurred within 30 min after collection.

### Fresh semen evaluation

The fresh semen quality in terms of semen volume, progressive motility, and sperm concentration was conventionally evaluated according to Chauychu-noo et al [13]. Semen volume was recorded directly on the microtube with 1.5 mL. To determine the progressive motility, 5 μL of semen sample diluted with 45 μL of Schramm extender was estimated by microscopic observation at 400× magnification. A total of 200 sperm were counted in at least 5 microscopic fields to obtain the final reading. The progressive motility was presented in terms of motile sperm percentage. The sperm concentration was determined using a hemocytometer chamber. Five μL of semen sample was diluted with 995 μL of 4% sodium chloride. A drop of semen sample was put on a hemocytometer, and the reading was recorded under a compound microscope (×400 magnification). Sperm concentration was expressed as billion (10^9^) sperm cells/mL. Only semen samples with motility ≥80% and sperm concentration ≥3×10^9^ sperm/mL were used in the experiment.

### Experimental design

The experiment determined the optimal concentration and the effect of sericin. Treatments were classified into four groups by the levels of sericin supplementation, including 0%, 0.25%, 0.50%, and 0.75%, which were added to the Schramm extender. The experiment was replicated five times. Post-thawed semen was evaluated for sperm motility, sperm viability, and lipid peroxidation. The fertility test in terms of percentages of fertility, hatchability on total eggs, and hatchability on fertile eggs was determined (4 replications).

### Semen dilution and cryopreservation

After fresh semen evaluation, the semen samples were pooled and diluted 1:3 (v:v) with a Schramm extender [14]. Then, the samples were cooled to 5°C for 45 to 60 min (1°C per 3 min) before the freezing procedure. DMF (N,N-dimethylformamide) was mixed with the samples to a final concentration of 6% (v/v). The semen samples were then loaded into 0.5 mL plastic straws, sealed with polyvinylpyrrolidone powder, and equilibrated at 5°C for 15 min. After equilibration, the semen straws were cryopreserved using the liquid nitrogen (LN_2_) vapor method; the straws were laid horizontally on a rack 11 cm and 3 cm above the surface of LN_2_ (−35°C and −135°C) for 12 min and 5 min, respectively. Finally, the straws were plunged into LN_2_ and stored until analysis. The Styrofoam box (28×38×29 cm) was filled with 6.5 L of LN_2_.

Thawing of frozen semen was achieved at 5°C for 5 min. The freezing and thawing protocols were conducted according to our previous study [2].

### Frozen-thawed semen evaluation

#### Sperm motility

Total sperm motility and progressive motility were assessed using a computer-assisted sperm analysis system (version 12 TOX VIOS; Hamilton Thorne Biosciences, Beverly, MA, USA) with Olympus software to process video material recorded in the “avi” format. For each assessment, 5 μL of frozen-thawed semen sample was dropped into a prewarmed (37°C) counting chamber. Evaluations of at least five fields with a minimum of 300 sperm per sample were performed. Total motility expressed the percentage of sperm making any sort of movement. Progressive motility was expressed in sperm that were swimming in a straight line.

#### Sperm viability

The sperm viability was evaluated via dual fluorescent staining using SYBR-14 and propidium iodide kits (Live/dead sperm viability kit L7011; Invitrogen, Thermo Fisher Scientific, Waltham, MA, USA). Staining was performed according to the manufacturer’s instructions. Briefly, each sample was diluted to a concentration of 150 million sperm/mL, and 300 μL of diluted semen was mixed with 5 μL of SYBR-14 solution diluted in sterile water (at a ratio of 1:49) in a cytometric tube. The cells were then incubated at 5°C for 10 min, followed by staining with 5 μL propidium iodide for 5 min. The cells were then fixed with 30 mL of 10% formaldehyde. For assessment, at least 300 sperm cells were analyzed under an IX71 fluorescence microscope (Olympus, Tokyo, Japan) at 400× magnification. The nucleus of live sperm with an intact plasma membrane is stained bright green with SYBR-14, while that of dead sperm or sperm with a damaged plasma membrane is stained red with propidium iodide. Sperm viability was expressed as the percentage of live sperm with intact plasma membranes.

#### Lipid peroxidation

The concentration of MDA is an index of lipid peroxidation in semen samples, which can be measured using the thiobarbituric acid (TBA) reaction, according to Chankitisakul et al [15]. Semen samples were added to 0.25 mL of ferrous sulfate (0.2 mM) and 0.25 mL of ascorbic acid (1 mM), and they were then incubated for 60 min in a 37°C water bath. Next, the samples were added to 1 mL of trichloroacetic acid (15% [w/v]) and 1 mL of TBA (0.375% [w/v]) before boiling for 10 min. The samples were cooled to 4°C to stop the reaction. Finally, the samples were centrifuged at 800×g for 10 min at 4°C. Supernatants (2 mL) were used for analysis using a UV–Visible spectrophotometer (Analytikjena Model Specord 250 plus) at 532 nm.

#### Fertility test

The fertilizing test of the different treatments was tested by inseminating layer hens once a week with a dose of 0.4 mL of frozen-thawed spermatozoa with a final sperm concentration of 400×10^6^ sperm/hen from each group. Insemination was performed between 3:00 pm and 5:00 pm. Egg candling was performed to determine fertility on day 7 of incubation, and hatchability was determined on day 21 of incubation. Fertility was calculated as the percentage of fertile eggs in total eggs. The hatchability of total eggs was calculated as the percentage of hatched eggs of the total number of eggs. The hatchability of fertile eggs was calculated as the percentage of hatched fertile eggs.

### Statistical analysis

The data were analyzed by completely randomized design, and treatment groups were compared for differences using Tukey’s post hoc test. Before statistical analysis, the data were tested for normal distribution by the Shapiro–Wilk test and homogeneity of residual variances by Levene’s test, and outlier data were eliminated. The overall differences between treatment means were considered significant when p<0.05.

## RESULTS

### Fresh semen analysis

The average (±standard error [SE]) of progressive motility was 84.19%±0.81%. The means (±SE) semen volume and sperm concentration of fresh semen were 410±0.06 μL and 4.27±0.22×10^9^ spz/mL, respectively.

### Post-thaw semen analysis

The average (±SE) values for frozen-thawed semen quality are presented in Table 1. The total motility and progressive motility in 0.5% and 0.75% sericin groups were significantly higher than the 0% control group. Meanwhile, the highest viability was found in 0.50% sericin (p<0.05). A lower MDA level was found with 0.50% and 0.75% sericin supplementation than the 0% control group (p<0.05). Therefore, we inferred that 0.50% sericin supplementation was the optimal concentration.

The effects of different sericin levels on fertility and hatch ability are presented in Table 2. The fertility and hatchability of total eggs were significantly higher in the 0.50% sericin group than in the other groups (p<0.05). In addition, the hatchability of fertile eggs in the sericin group (0.25% to 0.75%) was higher than that in the control group (p<0.05).

## DISCUSSION

Sericin exhibits antioxidant activity by reducing lipid peroxidation resulting from free radicals [14]. A suitable dose of sericin addition in rooster semen freezing extenders must be considered, as it has been reported differently in each species [11,12,16]. We observed that supplementation of freezing extenders with 0.50% to 0.75% sericin showed greater total motility and progressive motility and lower MDA than other groups after cryopreservation. However, the viability of 0.75% decreased compared with 0.50%. Moreover, fertility ability was higher when 0.50% was added to the freezing extender.

The composition of the sperm membrane impacts the resistance to a decrease in temperature during the freezing process through its contributions to membrane fluidity and stability [17]. Greater degrees of cold shock mean greater damage to the sperm plasma membrane, which varies depending on the animal species as variations in the lipid composition of the C/PL ratio and PUFAs in the sperm plasma membrane [18,19]. Considering the proportion of (C/PL) for sperm plasma membrane from low to high were boar (0.26), rooster (0.30), stallion (0.36), and bull (0.45) [20]. A higher C/PL ratio allowed the membrane to remain fluid through the freezing procedure, resulting in good freezability. In the case of antioxidant supplementation, it is possible to infer that the optimal dose of antioxidant supplementation in each species was in accordance with the C/PL ratio. In bull sperm, which has a greater ratio (0.45) of cholesterol to phospholipids, an optimal dose of sericin was 0.25% [12], while 0.75% sericin seemed optimal for enhancing the sperm quality in boar semen [11], which has a low proportion of cholesterol to phospholipids (0.26). Similarly, in rooster sperm, which has a low proportion (0.30), supplementation of a freezing extender with 0.50% to 0.75% sericin showed greater total motility and progressive motility. These sperm qualities were in accordance with the concentration of MDA, which decreased with sericin supplementation (0.50% to 0.75%) compared with the control and 0.25%. It could be inferred that sericin could promote radical scavenging activity.

The toxicity of higher concentrations of sericin to cryo preserved sperm cells was identified in several studies. Kumar et al [16] and Yangngam et al [12] reported that 1% to 2% sericin had harmful effects on bull sperm cells; moreover, sericin supplementation at 1% in boar semen extenders was associated with reduced sperm quality [11]. In the present study, sericin supplementation at 0.75% did not have a helpful effect on sperm viability and fertility potential. This might be an imbalance between antioxidants and free radicals affected by the cell [21]. Excess antioxidant concentrations can cause hypertonic conditions, causing dehydration of sperm cells [22]. This could be explained by whether the viability of sperm cells in the 0.75% group decreased even though the MDA concentration tended to be lower than that in the 0.50% group. Therefore, selecting the appropriate sericin level for each animal species is an important factor in success.

In accordance with the frozen semen quality, the fertility obtained from insemination with frozen semen was significantly higher in the 0.50% sericin group than in the other supplemented groups and the control group; the hatchability of total eggs was in accordance with the fertility (Table 2). This fertility rate was better than that in numerous previous reports (10% to 40%) [23,24]. However, fertility was still lower compared with insemination using fresh semen. Additionally, some studies have reported greater fertility potential using semen cryopreserved above 80% [13,25]. The high variability in fertility potential in cryopreserved semen leads to difficulty in applying frozen semen in practice. The reason why freezing/thawing decreases the fertility rate of poultry semen is not completely understood. However, lower fertility using frozen semen with total motility of frozen-thaw semen between 57% and 65% was found whether Thai native hens were used to test fertility, not commercial hens, as reported by previous studies [13,25]. Therefore, it might be speculated that the breed of hen may affect the fertility rate, as Islam and Nishibori [26] suggested that exotic chickens show better fertility than indigenous chickens in an intensive system. In addition, the number of sperm cells stored in sperm storage tubules (SSTs) after intravaginal insemination with 100 to 200 million sperm cells was reported to be less than 2% [27], indicating that the insemination dose was not mainly affected by fertility [28]. In other words, the viable sperm after thawing were greater than the required sperm count and stored in SSTs. However, the characterization of SSTs in terms of the morphological and physiological features together with the number of resident sperm cells after insemination by frozen semen has never been determined whether those of poultry sperm cells continue to function or change.

Regarding the hatchability of fertile eggs, the difference in hatchability could be attributed to eggshell quality from different breeds. Any breed with thin eggshells results in reduced hatchability and weakened embryos [29]. Otherwise, for chickens at the same age that were reared under similar conditions, i.e., housing, rearing, and feeding together with the same incubator on the same days, the hatchability of fertile eggs should not be different. However, in the present study, the lowest hatchability of fertile eggs was found in the control, followed by the 0.75% group (Table 2), which was related to sperm quality in terms of viability. It is suggested that supplementation with an optimal dose of sericin in a freezing extender increased not only sperm quality but also embryo development, as similarly reported by Ghasemi et al [30].

## CONCLUSION

In conclusion adding, 0.50% sericin to the freezing extender improved frozen thawed semen quality and increased fertility potential in chickens.

## Figures and Tables

**Table 1:** Effects of different concentrations of sericin on total motility (MOT), progressive motility (PMOT), viability, and malondialdehyde (MDA) in frozen-thawed semen (mean±standard error)

Sericin levels	Parameters			

	MOT (%)	PMOT (%)	Viability (%)	MDA (μMol/mL)
0%	44.86±2.66^B^	17.03±0.64^B^	40.34±2.38^B^	3.50±0.81^A^
0.25%	52.71±2.65^AB^	20.82±2.80^AB^	46.64±2.51^AB^	3.44±0.11^AB^
0.50%	58.93±3.38^A^	26.38±2.46^A^	48.66±1.48^A^	3.21±0.17^B^
0.75%	63.12±3.30^A^	28.21±2.44^A^	41.94±2.74^B^	3.00±0.21^B^
p-value	0.0043	0.0124	0.0406	0.0440
F-value_(DF of treatment, DF of error)_	6.72_(3,16)_	5.11_(3,16)_	4.04_(3,16)_	3.66_(3,16)_

DF, degree of freedom.

A,B Different letters within columns indicate significant differences (p<0.05).

**Table 2:** Effects of different concentrations of sericin on fertility and hatchability in native hens (mean±standard error)

Sericin levels	Parameters		

	Fertility (%)	Hatchability on fertile eggs (%)	Hatchability on total eggs (%)
0%	36.41±0.68^B^ (88/239)	75.04±0.12^B^ (66/88)	27.62±1.27^C^ (66/239)
0.25%	33.45±0.15^B^ (92/240)	91.41±0.36^A^ (84/92)	35.01±1.38^B^ (84/240)
0.50%	54.92±0.50^A^ (148/265)	87.79±0.37^A^ (130/148)	49.12±2.26^A^ (130/265)
0.75%	37.39±0.36^B^ (80/228)	83.65±0.17^A^ (67/80)	29.25±1.16^C^ (67/228)
p-value	0.0159	0.0072	0.0252
F-value _(DF of treatment, DF of error)_	21.33_(3,12)_	37.13_(3,12)_	15.34_(3,12)_

DF, degree of freedom.

A–C Different letters within columns indicate significant differences (p<0.05).
